# International Medical Congress

**Published:** 1887-03

**Authors:** 


### ARTICLE V.

—THE NINTH—

### INTERNATIONAL MEDICAL CONGRESS,

WILL BE HELD AT

WASHINGTON, D. C., September 5th, 1887.

---

*President :*

N. S. DAVIS, M. D., LL. D.

---

*Secretary General :*

JNO. B. HAMILTON, M. D., of U. S. A.

---

### SECTION 17.

---

### *DENTAL AND ORAL SURGERY.*

*President :*

DR. J. TAFT.

---

*Vice-Presidents :*

Dr. W. W. Allport, 242 Wabash Ave., Chicago, Ills.
Dr. F. Abbott, 22 W. 40th St., New York City, N. Y.
Dr. W. C. Barrett, 208 Franklin St., Buffalo, N. Y.

Dr. S. W. Dennis, San Francisco, Cal.
Dr. C. L. Ford, Ann Arbor, Mich.
Dr. W. H. Morgan, Nashville, Tenn.
Dr. H. J. McKellops, 2630 Washington Ave., St. Louis, Mo.
Dr. A. T. Metcalf, Kalamazoo, Mich.
Dr. A. L. Northrop, 57 W. 49th St., New York City, N. Y.
Dr. A. O. Rawls, Lexington, Ky.
Dr. Joseph Richardson, Terre Haute, Ind.
Dr. C. W. Spalding, St Louis, Mo.
Dr. L. D. Shepard, 100 Boylston, St., Boston, Mass.
Dr. James Truman, 3249 Chestnut St., Philadelphia, Pa.
Dr. W. W. H. Thackston, Farmville, Va.
Dr. V. E. Turner, Raleigh, N. C.

*Foreign Vice-Presidents :*

Prof. Dr. F. Busch, Berlin, Germany.
Dr. W. Herbst, Bremen, Germany.
Dr. L. N. Hollander, Halle, Germany.
Dr. Andrieu, 2 Rue de le paix, Paris, France.
Dr. E. Magitot, Paris, France.
Dr. V. Haderup, Copenhagen, Denmark.
Dr. T. H. Harding, London, England.
Dr. W. George Beers, Montreal, Canada.

*Secretaries :*

Dr. E. A. Bogue, 29 E. 20th St., New York City, N. Y.
Dr. F. H. Rehwinkel, Chillicothe, O.
Dr. E. Brasseur, 6 Rue Mogador, Paris, France.
Dr. Elof Fœrberg, Stockholm.
Dr. Julius Parreidt, Leipzig, Germany.

*Council :*

Dr. R. R. Andrews, Cambridge, Mass.
Dr. C. W. Bœdecker, New York City, N. Y.
Dr. C. A. Brackett, Newport, R. I.
Dr. B. H. Catching, Atlanta, Ga.
Dr. George H. Chance, Portland, Ore.
Dr. E. S. Chisholm, Tuscaloosa, Ala.

Dr. C. C. Chittenden, Madison, Wis.
Dr. D. M. Clapp, Boston Mass.
Dr. W. R. Clifton, Waco, Tex.
Dr. J. S. Cassidy, Covington, Ky.
Dr. K. B. Davis, Springfield, Ills.
Dr. A. M. Dudley, Salem, Mass.
Dr. M. W. Foster, Baltimore, Md.
Dr. J. G. Freidrichs, New Orleans, La.
Dr. C. E. Francis, New York City, N. Y.
Dr. Geo. L. Field, Detroit, Mich.
Dr. F. J. S. Gorgas, Baltimore, Md.
Dr. P. G. C. Hunt, Indianapolis, Ind.
Dr. A. O. Hunt, Iowa City, Ia.
Dr. R. Findley Hunt, Washington, D. C.
Dr. George W. Keely, Oxford, O.
Dr. Edw. C. Kirk, 1602 Arch St., Philadelphia, Pa.
Dr. James Lewis, Burlington, Vt.
Dr. James McManus, Hartford, Conn.
Dr. W. N. Morrison, St. Louis, Mo.
Dr. J. Hall Moore, Richmond, Va.
Dr. T. T. Moore, Columbia, S. C.
Dr. Edgar Palmer, LaCrosse, Wis.
Dr. A. J. Plomteaux, San Francisco, Cal.
Dr. S. B. Palmer, Syracuse, N. Y.
Dr. W. A. Spalding, Minneapolis, Minn.
Dr. C. A. Stockton, Newark, N. J.
Dr. A. H. Thompson, Topeka, Kan.
Dr. W. C. Wardlaw, Augusta, Ga.
Dr. J. W. White, Philadelphia, Pa.

*Finance Committee of this Section.*

Dr. J. W. White, Treasurer, Cor. 12th & Chestnut St. Philadelphia, Pa.
Dr. A. M. Dudley, Secretary, Salem, Mass.
Dr. Edgar Palmer, LaCrosse, Wis.
Dr. H. J. MeKellops, 2630 Washington Ave. St. Louis, Mo.
Dr. L. D. Shepard, 100 Boylston St., Boston, Mass.

Dr. C. H. Winkler, Augusta, Ga.
Dr. W. W. Allport, 242 Wabash Ave., Chicago, Ills,
Dr. W. W. Walker, 67 W. 9th St., New York City, N. Y.

*Reception Committee of this Section.*

Dr. A. L. Northrop, 57 W. 49th St., New York City, N. Y.
Dr. H. J. McKellops, 2630 Washington Ave., St. Louis, Mo.
Dr. A. M. Dudley, Salem, Mass.
Dr. William Carr, 35 W. 46th St., New York City, N. Y.
Dr. L. D. Shepard, 100 Boylston St., Boston, Mass.
Dr. E. Maynard, Washington, D. C.
Dr. D. McFarlan, Washington, D. C.
Dr. James McManus, Hartford, Conn.
Dr. W. W. H. Thackston, Farmville, Va.
Dr. E. A. Bogue, 29 E. 20th St., New York City, N. Y.
Dr. F. H. Rehwinkel, Chillicothe, O.
Dr. C. F. W. Bœdecker, 60 E. 58th St., New York City, N. Y.

Permit the following suggestions. It will be the duty of the Reception Committee to receive and welcome the Foreign Guests and Visitors; and to facilitate their introduction to, and acquaintance with, the members of the Profession in this country, and to promote the social features of the occasion. It is hoped that, through this Committee, an acquaintance will be effected throughout the entire membership of the Section.

*Committee on Operative Dentistry and Oral Surgery—Clinics.*

Dr. C. F. W. Bœdecker, 60 E. 58th St., New York City, N. Y.
Dr. J. A. Watling, Ypsilanti, Mich.
Dr. R. L. Cochran, Burlington, Ia.
Dr. F. Abbott, 22 W. 40 St., New York City, N. Y.
Dr. W. C. Wardlaw, Augusta, Ga.
Dr. J. D. Patterson, Kansas City, Mo.

It will be the duty of this Committee to make ample provision for the work of this department, providing all needed facilities, such as chairs, engines and all appliances: to provide patients and subjects for the Clinics; to confer with the operators, learn their needs, and supply them so far as possible.

There will be others added to this Committee as the work may require.

*Committee on Prosthetic Dentistry.*

Dr. Geo. L. Field, Detroit, Mich.
Dr. H. B. Noble, Washington, D. C.
Dr. A. O. Hunt, Iowa City, Ia.
Dr. John Allen, New York City, N. Y.
Dr. T. T. Moore, Columbia, S. C.
Dr. W. N. Morrison, 1337 Washington Ave., St. Louis, Mo.
Dr. R. B. Donaldson, Washington, D. C.

It will be the duty of this Committee to provide facilities for the work of this department, and arrange for its efficient performance; and make such regulations as shall secure the greatest benefit from the demonstrations.

ORGANIZATION.

*The following rules and regulations have been adopted by the Ex-Committee, for the guidance of the work of the Congress, and of its sections.*

The Congress will consist of such members of the regular medical profession, as shall have registered, and taken their ticket of admission, and of such other scientific outmen, as the Executive Committee of the Congress shall deem it desirable to admit.

Books for registration of members, will be ready on and after September 1st, 1887, and on each subsequent day during the session. Any member desiring registration prior to this time, may apply by letter to the Secretary General, and forward his dues with his full address, when a receipt will be returned.

The membership fee, for residents of the United States, will be (10.00) ten dollars; there will be no dues for members from other countries. Each member will be entitled to receive a copy of the transactions of the Congress, when published by the Ex-Committee.

The work of the various sections will be directed, by the President of the section, and the order will be published in a daily programme for each Section.

Brief abstracts of papers to be read in the Sections, shall be fowarded to the Secretaries of the proper Section, on or before April 30th, 1887. These abstracts shall be treated as confidential communications. Papers relating to topics not included in the lists of subjects proposed, by the officers of the Sections, may be excepted after April 30th, 1887.

The officers of each Section shall decide as to the acceptance of such proposed communications, and the time for their presentation.

The Ex-Committee cordially invites members of the regular Medical profession, and men eminent of the *Sciences, collateral to Medicine, in all countries*, to participate in person, or by papers, in the work of this great humanitarian assembly.

The attendance of medical students, and others interested in the work of the various Sections, or in the general addreses delivered in Congress, will be permitted on the recommendation of the Secretary General, or by the officers of a Section, on their taking out of the registration committee a general ticket of admission fee, one dollar; such persons cannot take part in the proceedings.

All communications and questions relating to the special business of any section, must be addressed to the President, or one of the secretaries of that section.

*Officers of Sections, and Their Duties.*

The officers of each section, including foreigners shall shall be a *President*, not less than five *Vice Presidents*, four *Secretaries*, (two foreign) and not less than ten, nor more than thirty, members of council.

*President.*—The President of each section, shall be its executive officer, who is solely responsible for the efficient work of his section. He shall nominate all persons to the Ex-Committee, for any office connected with his section. He shall select and regulate (by conference where desired, with the other officers of his section), all papers or questions for discussion and reject only after such conference, such papers or questions, as he may deem inadmissible to the transactions, or for presentation in his section. He shall preside at, and regulate the business of each meeting of his section, punctually at the hour named, making an opening address to the section, if he so desires. He may at any time adjourn the session of the section, when in his opinion sufficiently long, as when there are too many papers for the day, etc., etc. He shall strictly enforce Rule 8 of the preliminary Organization. When a paper is read, or discussion occurs in a foreign language, he shall resign the chair to a foreign officer of the same nationality as the language employed.

*Vice-Presidents.*—They shall assist the President in the performance of his duties at each meeting of the Section when requested by him, and shall take their seats on each side of the presiding officer. They shall aid the President in consultation on the value and character of all papers or discussions that are to be presented in the Section or the transactions.

*Secretaries.*—The four Secretaries of Sections shall arrange among themselves, or at the request of the President of the Section, the order of their duties. They shall keep accurate records of the proceedings of each day in their Section, and make such daily report of it to the Congress, as the President of the Section shall direct; the foreign Secretaries acting for their own nationalities. At the close of the session they shall present all their minutes, in good order, to the Secretary General for publication in the Transactions, if so desired by the Executive Committee.

*Members of Council.*—It is expected that the Members of the Council will aid in every way to make the Sessions efficient and instructive. They will also be expected to advise with the President on any matter pertaining to the work of the Section, and on questions which may arise in connection with the publication, in the transactions of papers read, or discussion had in the Section.

